# Prognostic and Diagnostic Potential of the Structural Neuroanatomy of Depression

**DOI:** 10.1371/journal.pone.0006353

**Published:** 2009-07-27

**Authors:** Sergi G. Costafreda, Carlton Chu, John Ashburner, Cynthia H. Y. Fu

**Affiliations:** 1 Institute of Psychiatry, King’s College London, London, United Kingdom; 2 Wellcome Trust Centre for Neuroimaging, Institute of Neurology, University College London, London, United Kingdom; University of Muenster, Germany

## Abstract

**Background:**

Depression is experienced as a persistent low mood or anhedonia accompanied by behavioural and cognitive disturbances which impair day to day functioning. However, the diagnosis is largely based on self-reported symptoms, and there are no neurobiological markers to guide the choice of treatment. In the present study, we examined the prognostic and diagnostic potential of the structural neural correlates of depression.

**Methodology and Principal Findings:**

Subjects were 37 patients with major depressive disorder (mean age 43.2 years), medication-free, in an acute depressive episode, and 37 healthy individuals. Following the MRI scan, 30 patients underwent treatment with the antidepressant medication fluoxetine or cognitive behavioural therapy (CBT). Of the patients who subsequently achieved clinical remission with antidepressant medication, the whole brain structural neuroanatomy predicted 88.9% of the clinical response, prior to the initiation of treatment (88.9% patients in clinical remission (sensitivity) and 88.9% patients with residual symptoms (specificity), *p* = 0.01). Accuracy of the structural neuroanatomy as a diagnostic marker though was 67.6% (64.9% patients (sensitivity) and 70.3% healthy individuals (specificity), *p* = 0.027).

**Conclusions and Significance:**

The structural neuroanatomy of depression shows high predictive potential for clinical response to antidepressant medication, while its diagnostic potential is more limited. The present findings provide initial steps towards the development of neurobiological prognostic markers for depression.

## Introduction

While neurodegenerative disorders such as Alzheimer’s disease have diagnostic structural and functional brain abnormalities [1], the diagnosis of other psychiatric disorders is based entirely on clinical signs and symptoms. Investigation of objective, neurobiological markers would support diagnostic systems and treatment decisions. The potential of a biomarker though depends on its predictive power at the level of the individual.

We found that the *functional* neuroimaging correlates of core affective processing have significant potential as a diagnostic marker for depression. The functional neuroanatomy of implicit processing of sad facial expressions showed an accuracy of 86% in identifying individuals in an acute depressive episode [2], while verbal working memory had a more limited but still significant diagnostic accuracy [3]. Sad facial expressions are socially relevant, emotional cues which engage a distributed network of regions [4] that show an abnormal response during an acute depressive episode [5]–[6]. Moreover, the neural pattern to sad faces also demonstrated high prognostic potential for the prediction of clinical response to cognitive behavioural therapy (CBT) [7].

In the present study, we investigated the structural neuroanatomy of depression as a prognostic and diagnostic marker for depression. As a marker of clinical response in depression, we found that regional volumes in the anterior cingulate, temporal cortices and basal ganglia were correlated with the rate of clinical improvement [8]. The analysis though was limited to the original sample, and the predictive response in novel data was not explicitly examined. In schizophrenia, Davatzikos et al. [9] reported a diagnostic accuracy of 81% from whole brain structural neuroimaging features. However, global cerebral volume in major depression is comparable to healthy individuals, in contrast to schizophrenia [10]. Instead, structural deficits in depression appear to be more localised within a distributed pattern, which include the hippocampus [11], subgenual anterior cingulate [12]–[13], orbitofrontal and middle frontal cortices [14], and basal ganglia [reviewed in: 10], [15].

We expected the structural correlates of depression to show significant predictive potential for treatment with antidepressant medication, implicating regions which would include the anterior cingulate cortex, while the predictive potential for treatment with CBT was less clear. As a potential diagnostic marker, we expected a lower accuracy than observed in schizophrenia [8], which would encompass a distributed network including the anterior cingulate and prefrontal regions, hippocampus, and basal ganglia.

## Results

**Figure 1:**
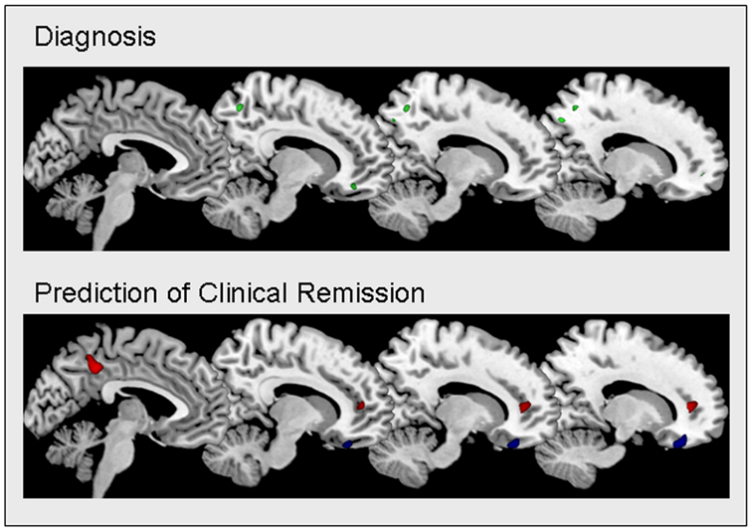
Sagittal cross-sectional view of regions pertinent for diagnosis and prediction of treatment response in depression. In the top panel, sagittal views are presented which show medial regions of decreased grey matter density which contributed to the diagnosis of depression (coloured in green) in the right subgenual anterior cingulate (BA 25) and precuneus (BA 7). No regions of increased grey matter in patients with depression relative to healthy individuals contributed to the diagnosis. In the lower panel, increased grey matter density in the anterior and posterior cingulate cortices (red) increased the probability of clinical remission to treatment with the antidepressant medication fluoxetine. Greater density in the orbitofrontal cortex (blue) increased the odds of residual symptoms of depression following antidepressant medication. Regions depicted were selected as relevant to the classification of patients as achieving remission or non-remission clinical status following fluoxetine treatment by every cross-validated support vector machine classification model. Sagittal views are presented in MNI space at z = −4, 10, 12 and 14.

The structural neuroanatomy of acutely depressed patients, before the initiation of treatment, correctly predicted clinical remission to treatment with the antidepressant medication fluoxetine with an accuracy of 88.9% (88.9% of patients in clinical remission (sensitivity) and 88.9% patients with residual symptoms (specificity), *p* = 0.01). Clinical remission was predicted by greater grey matter density in the right rostral anterior cingulate cortex (BA 32), left posterior cingulate cortex (BA 31), left middle frontal gyrus (BA 6), and right occipital cortex (BA 19) (Figure 1). Regions which predicted residual symptoms were the orbitofrontal cortices bilaterally (BA 11), right superior frontal cortex (BA 10) and left hippocampus. The structural neuroanatomy did not show a significant prediction of clinical remission to CBT.

As a diagnostic marker, the accuracy was 67.6% from whole brain structural neuroanatomy (64.9% patients with depression (sensitivity) and 70.3% healthy individuals (specificity), *p* = 0.027). Decreased grey matter density in the following regions showed the highest contribution to the diagnosis of depression: right subgenual anterior cingulate (BA 25), medial frontal gyrus (BA 11), superior temporal cortex (BA 22), precuneus (BA 7), hippocampus and thalamus, as well as in the left inferior parietal cortex (BA 40), occipital (BA 19) cortex, and cerebellum. No regions of increased grey matter in depressed patients relative to healthy individuals contributed to the diagnosis.

Regions which contributed to the prediction of treatment response were distinct from those relevant for diagnosis as there was no overlap anywhere in the brain between their respective brain patterns.

## Discussion

Whole brain structural neural correlates of depression identified 89% of patients who subsequently had a full clinical response to the antidepressant medication fluoxetine. The structural neuroanatomy of depression has significant potential as a prognostic marker of treatment response with antidepressant medication. In contrast, the structural neuroanatomy showed limited potential as a diagnostic measure for depression.

The findings support functional [5], [16]–[18] and structural [8] neuroimaging studies implicating the anterior cingulate cortex as a marker of clinical response to antidepressant medication, but also identified a more widespread network which included the posterior cingulate. The anterior and posterior cingulate cortices are strongly interconnected [19], and their functions are complementary with the anterior cingulate subserving executive functions linked to emotional and autonomic responses while the posterior cingulate has a more evaluative role that is postulated to direct activity in the anterior cingulate [20]. The data also point to a more widespread network of regions which are predictive of clinical response, including the hippocampus which may reflect stress-induced neuroplastic changes [21]–[24]. In particular, the present study suggests that grey matter density in a set of regions predicts how well an *individual* patient will respond to antidepressant treatment. In contrast, whole brain functional responses to sad faces showed high predictive potential to CBT treatment [6].

Regions important for individual diagnosis have been featured within the cortico-striato-pallido-thalamic loops, which include the medial and orbital prefrontal cortices, amygdala, hippocampus, medial thalamus, and striatum [25], and cortico-cortical circuits from the medial prefrontal cortex connecting the parahippocampus, posterior cingulate and superior temporal cortices [26]. In depression, volumetric and cellular deficits have most consistently been identified in the hippocampus [11], but as well in the anterior [12] and posterior cingulate [13], orbitofrontal [14], lateral temporal and occipital cortices [23], [27], and amygdala [28]. However, the structural neuroanatomy only showed limited potential for diagnosis, suggesting that structural abnormalities in depression are slight in contrast to other psychiatric disorders, such as schizophrenia [9]. Instead, functional brain activity to sad facial expressions may be a more accurate diagnostic marker of depression [2].

A limitation of the present study was the small sample sizes in the prediction of clinical response, which may not have provided sufficient power to find an effect for CBT. Although such negative findings should be treated with caution, one interpretation would be that structural brain regions predictive of response to CBT, should they exist, may be more subtle than those predictive of fluoxetine response. Yet, as the sample for the CBT treatment group was sufficient to detect a predictive potential of functional MRI [7], it is possible that if structural effects exist, they might be more subtle than functional ones. Another limitation was that the pharmacological treatment was a single medication from the class of serotonergic reuptake inhibitors. The predictive potential for other antidepressant medications and from other classes requires further investigation. Moreover, the specificity of the predictive marker is somewhat equivocal as there was no placebo treatment arm. All patients in the present study were medication-free and suffering from an acute depressive episode at the time of the MRI scan. The generalisability of our findings to patients with more chronic forms of depression and the effects of medication from different classes, such as noradrenergic or combined noradrenergic and serotonergic mechanisms [18], require further investigation.

In summary, the structural neural correlates of depression show high prognostic potential for treatment with the antidepressant medication fluoxetine. However, the diagnostic accuracy with structural neuroanatomy was more limited, while greater diagnostic potential may be found with functional neural correlates. The present findings may provide an initial step towards developing personalised clinical treatment options.

## Materials and Methods

### Participants

All participants provided written informed consent in accordance with the guidelines of the Institute of Psychiatry and South London and Maudsley (SLAM) NHS Trust Ethics (Research) Committee. Patients were 37 right-handed individuals (mean age 41.9 years, SD 8.9; 28 women) meeting Diagnostic and Statistical Manual of Mental Disorder-IV (DSM-IV) criteria [29] for major depression by Structured Clinical Interview for DSM-IV [30], in an acute episode of moderate severity, having a minimum score of 18 on the 17-item Hamilton Rating Scale for Depression (HRSD) (mean HRSD 20.7, SD 2.2) [31]. Exclusion criteria were a history of neurological trauma resulting in a loss of consciousness, a current neurological disorder, history of diabetes or other medical disorder, other Axis I disorder including an anxiety disorder, history of substance abuse within 2 months of study participation, or an Axis II disorder. All patients were free of psychotropic medication for a minimum of 4 weeks at recruitment (8 weeks for fluoxetine) and patients in the CBT treatment group remained medication-free throughout the treatment. Healthy controls were 37 right-handed individuals matched for age, gender and IQ (mean age 42.2 years, SD 9.0; 28 women) with no history of a psychiatric disorder, neurological disorder or head injury resulting in a loss of consciousness, and an HRSD score≤7 (mean HRSD 0.2, SD 0.6). There was no significant difference in age between groups (paired t-test, t = 0.17, df = 36, p = 0.87) or verbal IQ: patients 109.6, controls 114.1 (paired t-test, t = 1.16, df = 25, p = 0.25). All participants were recruited by advertisement from the local community, and all patients were outpatients. Some of the patient group had participated in a treatment study of depression with the antidepressant medication fluoxetine 20 mg daily (18 depressed patients) [4] or with CBT (12 depressed patients) [8], in which clinical remission was defined as a HRSD≤7 following 8 weeks of treatment with fluoxetine (9 patients achieved remission, 9 with residual symptoms) or 16 weeks with CBT (6 remission, 6 residual symptoms) (Table 1). The remaining patients only participated in a single MRI scan and declined the longitudinal treatment study.

**Table 1:** Demographic features.

	Healthy Controls	Depressed Patients	Medication Treatment		CBT Treatment	
			Remission	Non-remission	Remission	Non-remission
Number of subjects	37	37	9	9	6	6
Mean Age (years)	42.8 (6.7)	43.2 (8.8)	44.2 (10.3)	44.1 (6.0)	41.2 (11.7)	42.7 (6.6)
Sex (m/f)	9/28	9/28	2/7	2/7	2/4	1/5
Verbal IQ	114.1 (13.0)	109.6 (17.1)	107.8 (13.0)	101.1 (13.2)	118.2 (16.4)	107.2 (24.4)
Baseline HRSD	0.2 (0.6)	20.6 (2.2)	20.2 (1.7)	22.0 (2.8)	20.7 (2.0)	20.8 (1.9)
Final HRSD	0.0 (0.0)	8.5 (4.8)	4.2 (1.5)	12.2 (4.4)	2.8 (2.8)	10.0 (5.7)

Remission was defined as a final HRSD≤7 after 8 weeks of treatment with fluoxetine or 16 weeks of treatment with CBT, and Non-remission was a final HRSD>7; HRSD: Hamilton Rating Scale for Depression; CBT: cognitive behavioural therapy.

### Image Acquisition

Structural magnetic resonance imaging (MRI) data were acquired as 3D spoiled gradient recalled (SPGR) T1-weighted scans on a 1.5 T GE NV/i Signa system (General Electric, Milwaukee, Wisconsin) at the Maudsley Hospital, SLAM NHS Trust, London. The acquisition parameters were: TE = 8, TR = 24 ms, flip angle = 30°, field of view = 25 cm×25 cm, slice thickness = 1.3 mm, number of slices = 124, image matrix = 256×256×124.

### Image Analysis

Voxel-based morphometry (VBM) was applied to the structural MRI images using SPM5 (Wellcome Trust Centre for Neuroimaging, UCL, London, UK). The images were segmented into grey matter (GM), white matter (WM) and cerebrospinal fluid and imported into a rigidly aligned space [32]. GM segments were then iteratively registered by non-linear warping to templates generated from all images in each group by the Diffeomorphic Anatomical Registration Through Exponentiated Lie algebra (DARTEL) toolbox [33]. Modulation with additional scaling by the Jacobian determinants of the nonlinear deformation was applied to the normalized images [34]–[35] to preserve the overall amount of each tissue class after normalisation. Images were smoothed with a 6 mm full width at half maximum (FWHM) Gaussian kernel. The outputs of this procedure were the population templates of GM and the deformation parameters of each individual to this template. The deformation parameters were then used to generate the modulated and normalized GM maps, which are in a standard space, and to conserve global GM volumes. The input features for the subsequent analysis were the smoothed modulated normalized GM images.

Given the very high dimensionality of the VBM output (thousands of voxels, or features, for each subject, each one corresponding to one dimension) and the expectation that only a few of these features would be meaningful for prediction, we applied a further feature selection step [36]. We used whole-brain ANOVA filtering to select the areas of maximum group differences between patients and controls. First the t-value and degrees of freedom were estimated for each voxel in the training set. Then the t-map was converted into a p-map, and voxels higher than the threshold (uncorrected *p* = 0.005) were masked out and discarded for classification purposes.

Support vector machine is a supervised, multivariate classification method [37] with optimal empirical performance in many classification settings [38] that has previously been utilized in neuroimaging research [2]–[3], [7], [9]. Supervised refers to the training step in which the differences between the groups to be classified are learned. With structural MRI data, individual images are treated as points located in a high dimensional space, defined by the GM voxel values of the ANOVA-thresholded maps. A linear decision boundary in this high dimensional space is defined by a hyperplane, and SVM finds the hyperplane that maximizes the margin between two training groups, i.e. the separation between the training subjects that are most ambiguous and difficult to classify. In the SVM classification, the whole multivariate VBM pattern over the set of thresholded areas jointly generated the significant classification results, and the significance of such results therefore refers to the whole pattern.

To examine whether the SVM classifier could be expected to predict diagnosis or prognosis in new patients, we trained the model with leave-one-out cross validation. For each cross validation iteration, the data were partitioned into training and test sets. A different participant from each group was excluded at each iteration, and the SVM classifier was trained on the data from the other subjects, after the ANOVA feature selection step. This classifier was then used to predict the status of the test participant based on their structural scan alone. The process was repeated leaving each participant out once, allowing an accuracy measure to be determined based on the number of test examples correctly classified. Statistical significance of the overall classification accuracy was determined by permutation testing, by repeating the cross-validation procedure 300 times with a different random permutation of the training group labels. The SVM classifier was implemented using freely available software (LIBSVM, http://www.csie.ntu.edu.tw/~cjlin/libsvm).
